# Transcriptome of Cultured Lung Fibroblasts in Idiopathic Pulmonary Fibrosis: Meta-Analysis of Publically Available Microarray Datasets Reveals Repression of Inflammation and Immunity Pathways

**DOI:** 10.3390/ijms17122091

**Published:** 2016-12-13

**Authors:** Laurent Plantier, Hélène Renaud, Renaud Respaud, Sylvain Marchand-Adam, Bruno Crestani

**Affiliations:** 1 Centre d’Étude des Pathologies Respiratoires-CEPR, Institut National de la Santé et de la Recherche Médicale-INSERM, Unité Mixte de Recherche-UMR1100, Labex Mabimprove, 37000 Tours, France; renaud.respaud@univ-tours.fr (R.R.); Sylvain.marchand-adam@univ-tours.fr (S.M.-A.); 2 Université François Rabelais, 37000 Tours, France; 3 Centre Hospitalier Régional Universitaire-CHRU de Tours, Hôpital Bretonneau, Service de Pneumologie et Explorations Fonctionnelles Respiratoires, 37000 Tours, France; 4 Institut National de la Santé et de la Recherche Médicale-INSERM, Unité Mixte de Recherche-UMR1152, Labex Inflamex, 75018 Paris, France; helene.renaud@etu.univ-tours.fr (H.R.); bruno.crestani@aphp.fr (B.C.); 5 Centre Hospitalier Régional Universitaire-CHRU de Tours, Hôpital Trousseau, Service de Pharmacie, 37170 Chambray-les-Tours, France; 6 Université Paris Diderot, PRES Sorbonne Paris Cité, 75018 Paris, France; 7 AP-HP, Hôpital Bichat, Service de Pneumologie A, DHU FIRE, 75018 Paris, France

**Keywords:** fibroblasts, myofibroblast, pulmonary fibrosis, differentiation, microarray

## Abstract

Heritable profibrotic differentiation of lung fibroblasts is a key mechanism of idiopathic pulmonary fibrosis (IPF). Its mechanisms are yet to be fully understood. In this study, individual data from four independent microarray studies comparing the transcriptome of fibroblasts cultured in vitro from normal (total *n* = 20) and IPF (total *n* = 20) human lung were compiled for meta-analysis following normalization to z-scores. One hundred and thirteen transcripts were upregulated and 115 were downregulated in IPF fibroblasts using the Significance Analysis of Microrrays algorithm with a false discovery rate of 5%. Downregulated genes were highly enriched for Gene Ontology and Kyoto Encyclopedia of Genes and Genomes (KEGG) functional classes related to inflammation and immunity such as Defense response to virus, Influenza A, tumor necrosis factor (TNF) mediated signaling pathway, interferon-inducible absent in melanoma2 (AIM2) inflammasome as well as Apoptosis. Although upregulated genes were not enriched for any functional class, select factors known to play key roles in lung fibrogenesis were overexpressed in IPF fibroblasts, most notably *connective tissue growth factor* (*CTGF*) and *serum response factor* (*SRF*), supporting their role as drivers of IPF. The full data table is available as a supplement.

## 1. Introduction

Multiple mechanisms affecting many resident cell types of the lung participate in the initiation and progress of fibrosing lung disease such as idiopathic pulmonary fibrosis (IPF). Among these mechanisms, pro-fibrotic activation of lung fibroblasts is of particular interest because this gain-of-function phenomenon lends itself to therapeutic intervention. This aspect is illustrated by the beneficial effects of small molecules such as pirfenidone and nintedanib, which both block activation of lung fibroblasts in vitro [1,2]. Improved understanding of the molecular mechanisms driving fibroblast activation in IPF is the key to further therapeutic progress in this field.

Activation of lung fibroblasts in IPF is multifactorial and involves both alterations of the lung microenvironment and heritable cell-autonomous determinants. In support of the latter, IPF lung fibroblasts show phenotype changes in contrast with fibroblasts cultured from normal lungs, including expression of contractile proteins and extracellular matrix components such as collagen-1 [3]. These phenotype changes are maintained after the cells are passaged in vitro, and are sufficient to induce lung fibrogenesis when the cells are adoptively transferred into mice [4]. Heritable differentiation of lung fibroblasts may thus contribute to explain the intractable progression of IPF.

Identification of differentially expressed genes in cultured IPF lung fibroblasts may provide key insight into the mechanisms of fibroblast activation in IPF. To this aim, multiple experiments comparing the transcriptome of IPF lung fibroblasts to normal lung fibroblasts using RNA microarrays were previously published [5,6,7,8]. These studies yielded conflicting results with little overlap [9], most likely because of the generally small study size, of the high variability inherent to clinical samples, and of possible center-related bias. We hypothesized that combining these individual studies into a single meta-analysis may allow for the identification of robust alterations of mRNA expression in IPF fibroblasts, with the potential to reveal or confirm pathogenic mechanisms and therapeutic targets.

## 2. Results

Preprocessed and normalized data from the four published microarray studies comparing the transcriptome of explant-cultured IPF lung fibroblasts to normal lung fibroblasts (GSE1724, GSE10921, GSE40839 and GSE44723) [5,6,7,8] were downloaded from the National Center for Biotechnology Information Gene Expression Omnibus website [10]. Technical aspects for each dataset are summarized in Table 1. For each dataset, the expression values of each oligonucleotide probe were transformed to z-scores.

The meta-analysis included 20 IPF fibroblast cultures and 20 control cultures. Expression data were available for 17,414 distinct transcripts. The full dataset is available as Supplementary Table S1. Since data were not available for all genes in all datasets, a number of data points were missing. Full data (*n* = 20 Controls and *n* = 20 IPF) were available for 4238 genes (24% of the full dataset). Comparison of expression levels identified 115 mRNAs that were expressed at higher levels in IPF fibroblasts, and 113 downregulated genes, all listed in Supplementary Table S2. Table 2 shows the 10 most significantly upregulated and downregulated mRNAs in IPF fibroblasts.

**Figure 1:**
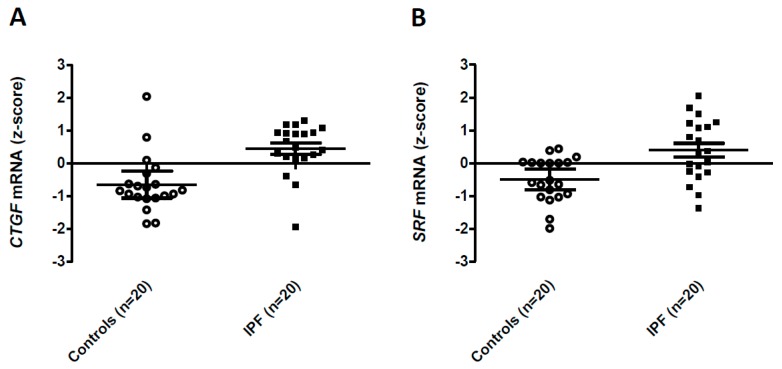
Expression levels of CTGF (**A**) and SRF (**B**) in Control and IPF fibroblasts. Data were obtained from the Gene Expression Omnibus GSE1724, GSE10921, GSE40839 and GSE44723 dataset and transformed to z-scores. Expression of *CTGF* and *SRF* mRNAs is shown as individual values, medians and 95% confidence intervals.

Among the 228 differentially expressed transcripts, 49 were previously associated with fibrogenesis in the biomedical literature (Table 2 and Supplementary Table S2). Several of these transcripts were among the 10 most significantly upregulated (LIMS2, NREP, CTGF) or downregulated (IL1R1, IFI44, NFKBIA) in IPF fibroblasts. Of particular interest, two factors with prominent roles in lung fibrogenesis, CTGF (Figure 1A) and Serum Response Factor (SRF, expression level +1.09 SD, corrected *p* value= 0.03, Figure 1B), featured among the upregulated transcripts in IPF fibroblasts.

Enrichment in genes putatively regulated by the microRNA miR-153 was observed among upregulated transcripts (13 genes out of 153 in the annotation: *ABAT*, *FAM168B*, *PPRC1*, *DVL3*, *ZMIZ1*, *MFAPL3*, *SLC38A1*, *XYLT1*, *ARL4A*, *ADO*, *FAM168A*, *PLEKHA3*, *AUTS2*, *p* = 1.2 × 10^−2^). Functional annotation analysis did not identify enrichment of Gene Ontology, pathway, functional class nor transcription factor binding site categories among transcripts upregulated in IPF fibroblasts.

**Figure 2:**
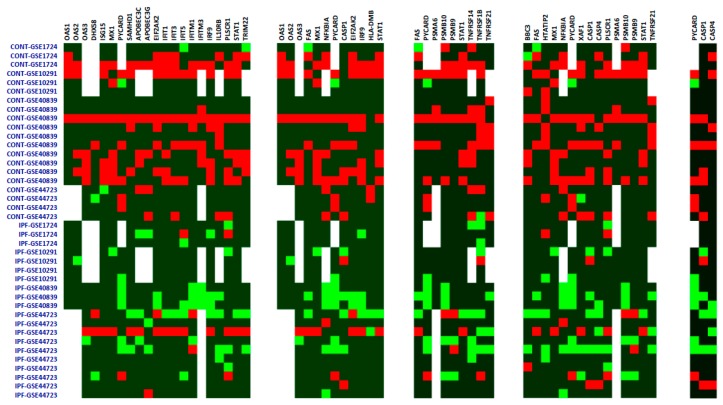
Expression levels of genes belonging to differentially expressed ontology and functional categories. Expression z-scores are shown as a heat map, with red showing increased expression and green showing lower expression. White denotes a missing value. Samples are identified as either Control (CONT) or IPF, followed by their dataset of origin. (**A**) GO-Defense response to virus; (**B**) KEGG-Influenza A; (**C**) GO-TNF mediated signaling pathway; (**D**) GO-Apoptotic process; (**E**) GO-AIM2 inflammasome. GO: Gene Ontology. KEGG: Kyoto Encyclopedia of Genes and Genomes. AIM2: Absent in melanoma.

Among the 10 most significantly down-regulated genes (Table 2) were several genes related to the immune and inflammatory response, such as interferon induced protein with tetratricopeptide repeats 1 (*IFIT1*), the interleukin 1 receptor, type I (*IL1R1*), and NFκB inhibitor α (*NFKBIA*). Analysis of the downregulated gene list yielded enrichment of five functional annotation clusters, which were related to the inflammatory/immune response and apoptosis (Table 3). Expression levels of genes belonging to the deregulated functional classes are shown in Figure 2.

Consistent with repression of processes involved in the immune/inflammatory response, the downregulated list was enriched with genes bearing binding sites for the Interferon Response Factor (IRF) transcription factor (11 genes out of 158 in the annotation: *DHX58*, *IDO1*, *IFI44*, *CASP1*, *PSMB9*, *PSMB10*, *SLC15A3*, *UBA7*, *ASPA*, *SLC12A7*, *TAP1*, *p* = 4.2 × 10^−6^) and the *Interferon Consensus Sequence Binding Protein* (*ICSBP*) transcription factor (9 genes out of 203 in the annotation : *IDO1*, *IFI44*, *PSMB9*, *SLC15A3*, *IFI35*, *UBA7*, *ASPA*, *SLC12A7*, *TAP1*; *p* = 3.2 × 10^−3^). No enrichment in putative microRNA targets was observed among downregulated genes.

**Figure 3:**
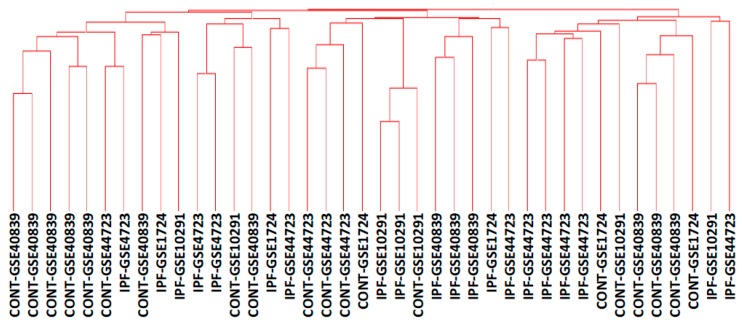
Unsupervised clustering of fibroblast cultures based on the full 17,414 genes list. The dendrogram results from Unweighted Pair Group Method with Arithmetic Mean (UPGMA) hierarchical clustering using all 17,414 genes in the dataset, using Pearson’s correlation as the similarity measure. Samples are identified as either Control (CONT) or IPF, followed by their dataset of origin.

Despite the identification of differentially expressed genes, functional classes and microRNA or transcription factor targets in IPF fibroblasts, unsupervised clustering did not allow for the separation of control and IPF fibroblast populations, as shown in Figure 3.

Because full data were available for only 24% of genes in the meta-analysis, an important question to answer was whether missing data impacted the global results of the study. We thus used SAM and the Database for Annotation, Visualization and Integrated Discovery (DAVID) to identify differentially expressed genes and functional annotation categories in IPF fibroblasts when analysis was restricted to genes without missing data (*n* = 4238). In the restricted analysis, 118 genes were significantly upregulated, and 72 genes were significantly downregulated in IPF fibroblasts. 48% of the genes upregulated in the whole dataset were upregulated in the restricted analysis, including *NAP1L3*, *KIAA0355*, *ASB1*, *EIF1*, *CTGF*, and *SRF*. 47% of the genes downregulated in the whole dataset were downregulated in the restricted analysis, including *IFIT1*, *PLSCR1*, *IL1R1*, *IFI44*, and *HTATIP2*. Similar to what was observed in the full dataset, there was no significantly enriched functional class among upregulated genes in the restricted analysis, while the most significantly enriched functional classes among downregulated genes were Immune Response, Influenza A, and Proteasome. These results were thus overall similar to those obtained with the full dataset.

## 3. Discussion

The main results of this study are that (1) select transcripts associated with lung fibrosis were upregulated in IPF fibroblasts; and (2) that the predominant whole-transcriptome alteration in IPF fibroblasts was the repression of interferon-driven viral defense programs.

Nucleosome assembly protein 1 like (*NAP1L3*) was the transcript most significantly upregulated in IPF fibroblasts. *NAP1L3* is part of nucleosome assembly complex and, therefore, participates in chromatin compaction. It is highly expressed in brain and nerve tissue, but also in lung at lower levels. Due to its function in chromatin organization, it may be speculated that *NAP1L3* participates in epigenetic control of gene expression by histone acetylation, in analogy with *NAP1L2* [17]. Perhaps more relevant to the present study, several transcripts previously associated with tissue fibrosis were upregulated in IPF fibroblasts. For instance, LIM zinc finger domain containing 2 (*LIMS2*) and neuronal regeneration related protein (*NREP*) were among the most significant genes. LIMS2 is a nuclear and focal adhesion protein that complexes with the integrin-linked kinase and represses cell spreading and migration. LIMS2 also localizes in the nucleus [18]. Double inactivation of *LIMS1* and *2* leads to the development of heart failure and fibrosis in mice [11]. *NREP* regulates the TGF-β signaling pathway, and overexpression of *NREP* in fibroblasts suffices to induce proliferation and myofibroblastic transformation [12]. *NREP* is upregulated in hypertrophic skin scars [19], suggesting that this protein may be important for fibrogenesis in vivo. Functional studies will be required to determine whether these proteins participate in lung fibrogenesis.

*CTGF* and *SRF*, two genes quite strongly associated with lung fibrosis in previous studies, were upregulated at the mRNA level in IPF fibroblasts in the present meta-analysis. CTGF, a member of the CCN protein family, is a highly potent extracellular matrix-associated mitogen. In vitro, *CTGF* induces collagen-1 expression in lung fibroblasts [20]. In vivo, overexpression of *CTGF* in fibroblasts drives fibrogenesis in multiple organs including the lung [21]. CTGF is overexpressed in IPF, with expression localized to alveolar epithelial cells and fibroblasts [22]. Consistent for a role of *CTGF* in IPF, a recent open-label uncontrolled study suggested that a monoclonal antibody targeting CTGF, FG-3019, may be beneficial in a subset of IPF patients in terms of lung function decline and imaging-assessed fibrotic changes [23]. SRF is a transcription factor that is activated by the p38 MAP kinase pathway [24] and by increased extracellular matrix stiffness through mechanotransduction [25]. *SRF* is essential for collagen-1 expression by lung fibroblasts in vitro [26], while inhibition of the *SRF* pathway by the small molecule CCG-203971 attenuates lung fibrogenesis in the bleomycin-induced lung fibrosis model in mice [27]. Interestingly, *SRF* drives *CTGF* expression in fibroblasts [28], raising the hypothesis that the stable overexpression of the *SRF/CTGF* axis may contribute to the heritable profibrotic phenotype of lung fibroblasts in IPF.

Despite increased expression of select transcripts associated with fibrotic processes in IPF fibroblasts, such alterations did not coalesce into coordinated changes as assessed by functional annotation analysis. By contrast, multiple functional classes were significantly enriched among downregulated genes. These were dominated by classes associated with inflammation and immunity, such as Defense response to virus and Influenza A defense programs, the TNF-α mediated signaling pathway, and the AIM2 inflammasome. Consistent with alteration of interferon-driven pathways, downregulated genes were enriched in *IRF* and *ICSBP* target genes.

Interactions between inflammation and immunity pathways and pro-fibrotic pathways are complex and context-dependent. Cytokines such as IL1-β, CCL2 and TNF-α induce collagen-1 expression in lung fibroblasts [29]. On the other hand, interferons inhibit growth of proliferating fibroblasts [30], while interferon-γ suppresses collagen-1 expression in lung fibroblasts both spontaneous [31] and induced by interleukin-1 [32], and inhibits TGF-β signaling by STAT1-dependent mechanisms [33]. Inflammation and immunity pathways may have protective, antifibrotic roles in IPF. An inactivating polymorphism in the Toll Like Receptor 3 gene, which is a key initiator of anti-viral defense systems, is associated with increased lung function decline and mortality in IPF [34]. This hypothesis is further supported by experimental and clinical data. *Tlr3^−/−^* knockout mice have increased fibrosis and reduced survival following bleomycin-induced lung injury, while fibroblasts bearing the *TLR3* Leu412Phe polymorphism have reduced NF-κB activation and keep proliferating in spite of treatment with the TL3 agonist poly(I:C) [34]. The PANTHER trial demonstrated increased mortality in IPF patients treated with corticosteroids and the immunosuppressive azathioprine [35]. Whether alteration of interferon-driven pathways in IPF can be addressed therapeutically is unclear in light of the failure of two clinical trials of interferon-γ [36,37].

The lack of a distinct functional or transcriptional mRNA overexpression signature in IPF fibroblasts suggests that post-transcriptional mechanisms may play key roles in the acquisition of the pro-fibrotic phenotype. Indeed, annotation analysis of overexpressed transcripts identified enrichment in miR-153 target genes in IPF fibroblasts in our study, suggesting reduced activity of this microRNA. Of interest, previous studies identified miR-153 as a potential endogenous repressor of lung fibrogenesis. miR-153 expression is reduced in the lungs of mice with bleomycin-induced fibrosis, while knockdown of miR-153 potentiates phosphorylation of Mothers against decapentaplegic homolog (SMAD)2/3 and myofibroblastic transformation induced by TGF-β in a human fetal lung fibroblast cell line [38]. Other micro-RNAs were implicated in the development of fibrotic lung disease [39]. Other alterations of the post-transcriptional regulation of gene expression associated with IPF fibroblasts are alterations in mRNA splicing and mRNA trafficking to ribosomes [40,41]. Additional screening studies relying on post-genomic information such as proteomics or metabolomics will be required to fully elucidate the mechanisms of fibroblast profibrotic differentiation in IPF.

The meta-analysis approach offers unique advantages for the global interpretation of gene expression changes reported in the original studies. In particular, the number of samples in the four original studies was low, thus their compilation allowed to gather a meaningful sample size. In addition, the meta-analysis approach reduced center-specific biases related to subject selection, lung sampling, fibroblast culture, and choice of the microarray platform. Technical and methodological issues must be kept in mind when analyzing the present results. Notably, identification of differentially expressed hits on high-throughput experiments usually relies on filtering based on excluding (1) genes with very low levels of expression and (2) genes with low fold-changes of expression, which are then considered to be of no biological significance. Because z-score normalization implied the loss of absolute expression values, it was not possible to identify genes with the highest or lowest level of expression. Full data was available for a fraction of the genome, although omitting genes with incomplete data did not impact the overall message of the analysis. The results presented here are descriptive, and were not verified experimentally by other techniques such as reverse transcriptase polymerase chain reaction. Transcriptome changes in IPF were also assessed in uncultured fibroblasts which were analyzed immediately after enzymatic dissociation of the lungs [42]. Because the present study was focused on heritable changes in gene expression, the corresponding dataset, where gene expression likely reflected microenvironmental cues, was not included. It cannot be ruled out whether a lack of power of the meta-analysis related to the high variability of microarray experiments led to relevant alterations of the transcriptome being missed from our analysis. Indeed several mRNAs shown to be expressed at higher levels in IPF fibroblasts, such as collagen-1, fibronectin-1 or α-smooth muscle actin [3], were not identified as overexpressed in the present study. It must be raised, however, that the number of samples in the current meta-analysis was quite higher than in any single-dataset study into IPF fibroblast mRNA expression published so far. Up-regulation of IFN regulated genes was observed in the lung of patients with SSc associated interstitial lung disease [15]. Because the corresponding GSE40839 study provided three IPF samples and 10 control samples out of 20 in our dataset, it may be raised that bias towards increased activation of inflammation and immunity pathways in the controls may have driven the results in the larger analysis. A likely explanation may be that heritable alterations in systemic sclerosis associated lung fibrosis are somewhat similar to IPF [43].

## 4. Materials and Methods

### 4.1. Data Compilation

All data used for the meta-analysis were processed and normalized data downloaded from GEO as provided in the “series matrix” files. Probe values were averaged into a single gene value when multiple probes were available for individual genes before transformation to z-scores [44]. In each dataset, the expression values of each gene were transformed to z-scores (z-score = individual gene value-mean gene value/standard deviation of all values for the gene). Z-scores were computed by rows (i.e., normalizing within the same gene expression). Thus, a total number of 17,414 genes were considered for analysis. Available gene expression data for each individual fibroblast culture were then compiled into a single spreadsheet. Since data were not available for all genes in all datasets, the corresponding data points were missing. Because all data were transformed to z-scores, it was not possible to calculate fold-changes in expression. The complete data table is available as a Supplementary Table S1.

### 4.2. Statistical Analysis

Variations in gene expression was defined by the difference between the z-scores of IPF and control samples. The identification of mRNAs differentially expressed in IPF versus control fibroblasts was performed using the web-enabled Significance Analysis of Microarrays tool [45], with a False Discovery Rate set to 5%. Z-score normalization has been shown to be suitable for the integration of microarray datasets [46]. Also, SAM has been shown to be suitable for meta-analysis of microarray datasets [47]. The SAM algorithm computes a *t*-test for each gene, and assesses the strength of the significance by permutation. SAM does not require any assumption on the distribution of the data, so it is correct to use this method on z-score data. This method was showed to perform well for microarray analysis [48]. The mean difference in transcript expression between IPF and control fibroblasts was expressed in standard deviations of the mean (SD). To identify differentially expressed transcripts with a putative participation in fibrogenesis, a review of the abstracted literature was performed with Pubmed [49] using the gene symbol and “fibrosis” and “not cystic fibrosis” as keywords. The presence of an association with fibrosis and a relevant reference were presented in the tables.

Clustered gene ontology categories and pathways enriched in the differentially expressed genes were identified with the DAVID v6.8 online tool [50,51] using Gene Ontology (GO) and Kyoto Encyclopedia of Genes and Genomes (KEGG) annotation. The program computed the probability that a GO or KEGG term was overrepresented by comparing the proportion of genes in the whole genome which are part of that GO term, to the proportion of the differentially expressed genes which are part of the same GO term using a modified Fisher’s exact test. Overrepresented GO or functional class terms were considered significantly overrepresented if (1) they contained a minimum of five relevant genes; (2) the *p* value was <0.05 following Benjamini-Hochberg (B&H) correction for multiple comparisons; and (3) the enrichment score was >2. Each cluster was described by the constitutive GO term name with the highest number of gene terms. To assess whether altered gene expression in IPF fibroblasts was consistent with altered transcription factor or microRNA activity, over and underexpressed gene lists were analyzed by interrogating the TargetScan and MSigDB databases using the TOPPFUN online tool [52,53] with a *p* value <0.05 following B&H correction. Finally, to assess whether mRNA expression profiles segregated IPF fibroblasts from control fibroblasts, hierarchical clustering analysis using Pearson’s correlation coefficients was performed with Hierarchical Clustering Explorer v3.5 (University of Maryland, College Park, MD, USA) [54].

## 5. Conclusions

Whether heritable profibrotic differentiation of lung fibroblasts in IPF results from genetic, epigenetic, transcriptional or post-transcriptional alterations is an important question for the development of novel therapeutics targeting these cells. The present results raise the hypothesis that downregulation of interferon-driven inflammatory and immune response pathways may participate in the profibrotic phenotype. In addition, the data are consistent with key roles of CTGF and SRF in fibroblast activation in IPF. Future studies at the protein or metabolite levels may provide further insight into the molecular mechanisms of cell-autonomous fibroblast activation in the fibrotic lung.

## Figures and Tables

**Table 1:** Technical characteristics of the source studies. N/A: not available. RMA: Robust Multi-array Average.

Dataset	Number of Culture Passages	Number of Samples	Microarray Platform	Number of Probes	Normalization Method
GSE1724	4–5	3 controls, 3 IPF	Affymetrix human U95Av2	12,625	N/A
GSE10921	4–7	3 controls, 4 IPF	Codelink Human Uniset I	10,353	Median
GSE44723	Up to 11	4 controls, 10 IPF	Affymetrix HG-U133 plus 2.0	21,095	RMA
GSE40839	2–5	10 controls, 3 IPF	Affymetrix human U133Av2	22,215	Invariant set

**Table 2:** The ten most significantly upregulated and downregulated genes in IPF fibroblasts compared with control lung fibroblasts. Expression level was defined by z-score (IPF)–z-score (Controls). N/A: not available.

Gene Symbol	Full Gene Name	Expression Level (z-Scores)	Implication in Fibrogenesis
**Upregulated Genes**			
*NAP1L3*	*nucleosome assembly protein 1 like 3*	1.49	
*PTHLH*	*parathyroid hormone like hormone*	1.10	
*KIAA0355*	*N/A*	1.12	
*LIMS2*	*LIM zinc finger domain containing 2*	1.24	Heart [11]
*ASB1*	*ankyrin repeat and SOCS box containing 1*	1.16	
*HHAT*	*hedgehog acyltransferase*	1.32	
*EIF1*	*eukaryotic translation initiation factor 1*	1.04	
*PAWR*	*pro-apoptotic WT1 regulator*	1.15	
*NREP*	*neuronal regeneration related protein*	1.20	Lung [12]
*CTGF*	*connective tissue growth factor*	1.09	Multiple [13]
**Downregulated Genes**			
*TRANK1*	*tetratricopeptide repeat and ankyrin repeat containing 1*	−1.23	
*IFIT1*	*interferon induced protein with tetratricopeptide repeats 1*	−1.20	
*SLC15A3*	*solute carrier family 15 member 3*	−1.36	
*CPED1*	*cadherin like and PC−esterase domain containing 1*	−1.38	
*PLSCR1*	*phospholipid scramblase 1*	−1.13	
*IL1R1*	*interleukin 1 receptor. type I*	−1.12	Skin [14]
*IFI44*	*interferon induced protein 44*	−1.11	Lung [15]
*HTATIP2*	*HIV−1 Tat interactive protein 2*	−0.99	
*PLEKHA4*	*pleckstrin homology domain containing A4*	−1.20	
*NFKBIA*	*NFκB inhibitor α*	−1.22	Liver [16]

**Table 3:** Functional annotation of downregulated genes. *p* values are computed following Benjamini and Hochberg correction.

Category	Source	Genes in List/Genes in Annotation	*p* Value (B&H)
Defense response to virus	Gene Ontology	21/399	5.9 × 10^−18^
Influenza A	KEGG	12/100	9.8 × 10^−6^
TNF mediated signaling pathway	Gene Ontology	9/234	3.2 × 10^−4^
Apoptotic process	Gene Ontology	15/1314	1.5 × 10^−3^
AIM2 inflammasome complex	Gene Ontology	3/7	2.7 × 10^−2^

## Supplementary Materials

Supplementary materials can be found at www.mdpi.com/1422-0067/17/12/2091/s1.
