# Supplementary material for: Transcriptome of Cultured Lung Fibroblasts in Idiopathic Pulmonary Fibrosis: Meta-Analysis of Publically Available Microarray Datasets Reveals Repression of Inflammation and Immunity Pathways
Source: Int J Mol Sci. 2016 Dec 13;17(12):2091. doi: 10.3390/ijms17122091 (PMC5187891; doi:10.3390/ijms17122091)
Supplement: Supplementary file 1 [file ijms-17-02091-s001.zip › ijms-154969-Supplementary Materials/ijms-154969-supp.pdf]

Supplementary Materials: Transcriptome of Cultured Lung Fibroblasts in IDIOPATHIC Pulmonary Fibrosis: Meta-Analysis of Publically Available Microarray Datasets Reveals Repression of Inflammation and Immunity Pathways

Laurent Plantier, Hélène Renaud, Renaud Respaud, Sylvain Marchand-Adam and Bruno Crestani

Table S2. Differentially expressed genes. Gene ID, q-values, the differential expression expressed in Standard Deviations are provided. Previous association with fibrogenesis is indicated along with a relevant reference.

Upregulated Transcripts				
**Gene ID**	**q-Value (%)**	**Differential Expression (SD)**	**Involved in Fibrogenesis**	**Reference**
NAP1L3	0.00	1.49		
PTHLH	0.00	1.10		
KIAA0355	0.00	1.12		
LIMS2	0.00	1.24	Heart	[1]
ASB1	0.00	1.16		
HHAT	0.00	1.32		
EIF1	0.00	1.04		
PAWR	0.00	1.15		
NREP	0.00	1.20		
CTGF	0.00	1.09	Multiple including lung	[2]
FTSJ1	0.00	1.20		
TES	0.00	1.01		
FAM168A	0.00	1.06		
PDZD8	0.00	1.11		
ZMIZ1	0.00	1.09		
SIPA1L1	0.00	1.02	Liver	[3]
FSTL3	1.27	1.11	Heart	[4]
ADO	1.27	1.03		
XYLT1	1.27	1.09	Skin	[5]
STAT5B	1.27	0.78		
SLC38A1	1.27	0.93		
VDR	1.93	0.98	Multiple including lung	[6]
LIMK2	1.93	1.15	Genital	[7]
TUBB2A	1.93	1.00		
GLRX2	1.93	0.99	Heart	[8]
EEF1B2	1.93	1.70		
SERINC5	1.93	1.03		
CDH2	1.93	0.96	Multiple including lung	[9]
MFAP3L	1.93	0.87		
EIF3J	1.93	0.99		
TMEM90B	2.92	1.01		
KRT33B	2.92	1.01		
AUTS2	2.92	0.96		
CALB2	2.92	1.02		
DACT1	2.92	1.20		
HES1	2.92	0.85	Lung	[10]
GPX7	2.92	0.95		
PLEKHA3	2.92	1.17		

Table S2. *Cont.*

Upregulated Transcripts				
**Gene ID**	**q-Value (%)**	**Differential Expression (SD)**	**Involved in Fibrogenesis**	**Reference**
SLC25A32	2.92	1.00		
TEX2	2.92	1.08		
JMJD6	2.92	0.83		
ARL4A	2.92	1.46		
UBL3	2.92	0.92		
SLC1A4	2.92	0.92		
AMIGO2	3.31	0.82		
PPRC1	3.31	0.94		
GMPPB	3.31	0.97		
C19orf61	3.31	1.05		
PRSS3	3.31	0.98		
FERMT2	3.31	0.94		
CIB1	3.31	0.79	heart	[11]
SRF	3.31	0.89	Multiple including lung	[12]
FAM168B	3.31	0.85		
TTC3	3.31	0.88		
RSU1	3.31	0.86		
PLP2	3.31	0.88		
KLC1	3.31	0.75		
HSPB7	3.31	0.83	Muscle	[13]
EPRS	3.31	0.85		
ATP13A2	3.31	1.14		
TARS	3.31	0.99	Lung	[14]
MTCH1	3.31	1.23		
SNF8	3.31	1.32		
MGC4294	3.31	1.07		
FAM195B	3.31	1.00		
THUMPD2	3.31	1.14		
VLDLR	3.31	0.93		
SLC25A15	3.31	0.95		
SARS	3.31	0.76		
ERI3	3.31	0.85		
PDLIM3	3.31	0.91		
OR7E156P	3.31	1.02		
CHST11	3.31	0.92		
CHUK	3.31	0.89	Liver	[15]
FUBP3	3.31	0.89		
RBP1	3.31	0.77	Liver	[16]
MAPRE1	3.31	1.26		
BICD2	3.31	0.85		
WDR1	3.31	0.88		
CADM1	3.81	0.85	Lung	[17]
HDGFRP3	3.81	1.09		
NUAK1	3.81	0.87		
TUBB2A	3.81	1.58		
LOC285359	3.81	2.27		
ARFGAP1	3.81	0.92		
SLC19A2	3.81	0.92		
ETV5	3.81	0.84		
ATP6V0B	3.81	0.91		
CAMTA2	3.81	0.85		
TIMP3	3.81	0.83	Multiple including lung	[18]
ABAT	3.81	0.73		

Table S2. *Cont.*

Upregulated Transcripts				
**Gene ID**	**q-Value (%)**	**Differential Expression (SD)**	**Involved in Fibrogenesis**	**Reference**
CMPK1	3.81	0.99		
CCDC6	3.81	0.96		
PMEPA1	3.81	1.15		
SERP1	3.81	0.89		
TSTA3	3.81	0.75		
RBM3	3.81	0.92		
LRRC59	3.81	1.15		
FAM89B	3.81	0.72		
MSN	4.47	1.28	Multiple including lung	[19]
COTL1	4.47	2.36		
NTM	4.47	1.01		
KIAA1217	4.47	0.79		
DVL3	4.47	0.72		
CIAO1	4.47	0.78		
MST4	4.47	0.83		
STRAP	4.47	0.85		
TXNL4A	4.47	0.75		
AKTIP	4.47	1.06		
KCNK7	4.47	1.36		
SLC3A2	4.47	0.89		
PREB	4.47	1.02		
NT5DC2	4.47	0.96		
TRIB1	4.47	0.87		
ITGBL1	4.47	0.88		
**Downreguated Transcripts**				
**Gene ID**	**q-Value (%)**	**Differential Expression (SD)**	**Involved in Fibrogenesis**	**Reference**
TRANK1	0.00	−1.23		
IFIT1	0.00	−1.20		
SLC15A3	0.00	−1.36		
C7orf58	0.00	−1.38		
PLSCR1	0.00	−1.13		
IL1R1	0.00	−1.12	Skin	[20]
IFI44	0.00	−1.11	Lung	[21]
HTATIP2	0.00	−0.99		
PLEKHA4	0.00	−1.20		
NFKBIA	0.00	−1.22	Liver	[22]
MAT2B	0.00	−1.35		
GBP2	0.00	−1.11		
SAMHD1	0.00	−1.07		
SESN1	0.00	−1.15		
TNFAIP2	0.00	−1.07		
VWA5A	0.00	−1.06		
STAT1	0.00	−1.04	Multiple including lung	[23]
SECTM1	0.00	−1.09		
NINJ1	0.00	−1.21		
TRPA1	0.00	−1.12	Cornea	[24]
IDO1	0.00	−1.10		
IL15RA	0.00	−1.06	Liver	[25]
PROCR	0.00	−1.02	Multiple including lung	[26]
IFITM3	0.00	−1.63		
NFE2L3	0.00	−1.27		

Table S2. *Cont.*

Downreguated Transcripts				
**Gene ID**	**q-Value (%)**	**Differential Expression (SD)**	**Involved in Fibrogenesis**	**Reference**
TRIM21	0.00	−1.02	Systemic sclerosis	[27]
DNPEP	1.69	−0.99		
SLC12A7	1.69	−1.13		
VPS39	1.69	−1.02	Skin	[28]
SNX6	1.69	−1.56		
IFI35	1.69	−1.02		
TMEM140	1.69	−0.98		
LPAR6	1.69	−1.27		
TRAFD1	1.69	−1.02		
PYCARD	1.69	−1.07	Kidney	[29]
ATP2B1	1.69	−0.90		
IFITM1	1.69	−0.98	Liver	[30]
NECAP2	1.69	−1.02		
SLFN12	1.69	−1.03		
TNFRSF1B	1.69	−1.01		
BTN3A2	1.69	−1.05		
IFIT3	1.69	−0.97		
GSTK1	1.69	−1.28		
AKR1C3	1.69	−1.04	Skin	[31]
FTL	1.69	−1.80		
SGCE	1.69	−0.94		
BBC3	1.69	−0.94	Lung	[32]
ZNF395	1.69	−1.02		
MAN1A1	1.69	−0.88		
IL32	1.69	−1.23	Liver	[33]
MX1	1.69	−0.96	Liver	[34]
STOM	2.00	−1.01		
EIF2AK2	2.00	−0.92		
TRIM22	2.00	−0.96		
PSMB10	2.00	−1.02		
FAM110B	2.00	−1.04		
HLA-DMB	2.00	−0.80		
OAS2	2.00	−1.32		
S100A3	2.00	−0.92		
CASP1	2.00	−0.87	Multiple including lung	[35]
FAS	2.00	−0.86	Multiple including lung	[36]
RAB8B	2.00	−1.14		
RDH14	2.00	−1.27		
PSMA6	2.00	−1.14		
APOBEC3C	2.00	−0.99		
CXCL1	2.00	−0.92	Multiple including lung	[37]
UBA7	2.00	−0.92		
IL7R	2.00	−1.02		
CALHM2	2.00	−1.14		
ASPA	2.00	−1.20		
SDCBP	2.92	−1.13		
TNFRSF21	2.92	−1.02		
XAF1	2.92	−0.91		
TAP1	2.92	−1.50	Kidney	[38]
SCNN1B	2.92	−1.18		
OAS3	2.92	−0.94		
WTAP	2.92	−0.81		
APOBEC3G	2.92	−1.06		

Table S2. *Cont.*

Downreguated Transcripts				
**Gene ID**	**q-Value (%)**	**Differential Expression (SD)**	**Involved in Fibrogenesis**	**Reference**
HERC3	2.92	−0.92		
BCL6	2.92	−0.93		
IL10RB	2.92	−1.01	Skin	[39]
MME	2.92	−0.90		
CBR3	2.92	−0.95		
PKIG	2.92	−1.00		
BTN3A3	3.31	−0.90		
AKR1B1	3.31	−0.87		
OAS1	3.31	−1.26	Multiple including lung	[21]
IRF9	3.31	−0.90	Liver	[40]
CXCL2	3.31	−0.89	Multiple including lung	[41]
DOCK4	3.81	−0.85		
C14orf159	3.81	−0.91		
TDRD7	3.81	−0.84		
LY6E	3.81	−0.92		
GNAI1	3.81	−0.87		
CASP4	3.81	−0.83	Kidney	[42]
RTP4	3.81	−1.16		
PSMB9	3.81	−0.91	Kidney	[38]
ATG14	3.81	−1.33		
BLOC1S1	3.81	−0.92		
NFIB	3.81	−0.89		
PTGES	4.47	−0.84	Multiple including lung	[43]
C1RL	4.47	−1.00		
TNFRSF14	4.47	−1.03	Multiple including lung	[44]
ISG15	4.47	−0.85	Liver	[45]
DHX58	4.47	−0.75		
MYLIP	4.47	−0.87		
ARHGAP28	4.47	−1.04		
ECHDC3	4.47	−1.06		
NR1H3	4.47	−1.10	Heart	[46]
TRIAP1	4.47	−0.78		
RALGPS2	4.47	−1.13		
IFIT5	4.47	−0.80		
ANKFY1	4.47	−0.83		

References

1. Liang, X.; Sun, Y.; Ye, M.; Scimia, M.C.; Cheng, H.; Martin, J.; Wang, G.; Rearden, A.; Wu, C.; Peterson, K.L.; et al. Targeted ablation of PINCH1 and PINCH2 from murine myocardium results in dilated cardiomyopathy and early postnatal lethality. *Circulation* **2009**, *120*, 568–576.
2. Ihn, H. The role of TGF-β signaling in the pathogenesis of fibrosis in scleroderma. *Arch. Immunol. Ther. Exp.* **2002**, *50*, 325–331.
3. Marfà, S.; Morales-Ruiz, M.; Oró, D.; Ribera, J.; Fernández-Varo, G.; Jiménez, W. Sipa1l1 is an early biomarker of liver fibrosis in CCl4-treated rats. *Biol. Open* **2016**, *5*, 858–865.
4. Panse, K.D.; Felkin, L.E.; López-Olañeta, M.M.; Gómez-Salinero, J.; Villalba, M.; Muñoz, L.; Nakamura, K.; Shimano, M.; Walsh, K.; Barton, P.J.R.; et al. Follistatin-like 3 mediates paracrine fibroblast activation by cardiomyocytes. *J. Cardiovasc. Transl. Res.* **2012**, *5*, 814–826.
5. Faust, I.; Roch, C.; Kuhn, J.; Prante, C.; Knabbe, C.; Hendig, D. Human xylosyltransferase-I—A new marker for myofibroblast differentiation in skin fibrosis. *Biochem. Biophys. Res. Commun.* **2013**, *436*, 449–454.
6. Ramirez, A.M.; Wongtrakool, C.; Welch, T.; Steinmeyer, A.; Zügel, U.; Roman, J. Vitamin D inhibition of pro-fibrotic effects of transforming growth factor β1 in lung fibroblasts and epithelial cells. *J. Steroid Biochem. Mol. Biol.* **2010**, *118*, 142–150.

7. Song, S.H.; Park, K.; Kim, S.W.; Paick, J.-S.; Cho, M.C. Involvement of Rho-Kinase/LIM Kinase/Cofilin Signaling Pathway in Corporal Fibrosis after Cavernous Nerve Injury in Male Rats. *J. Sex. Med.* **2015**, *12*, 1522–1532.
8. Mailloux, R.J.; Xuan, J.Y.; McBride, S.; Maharsy, W.; Thorn, S.; Holterman, C.E.; Kennedy, C.R.J.; Rippstein, P.; deKemp, R.; da Silva, J.; et al. Glutaredoxin-2 is required to control oxidative phosphorylation in cardiac muscle by mediating deglutathionylation reactions. *J. Biol. Chem.* **2014**, *289*, 14812–14828.
9. Kasper, M.; Haroske, G. Alterations in the alveolar epithelium after injury leading to pulmonary fibrosis. *Histol. Histopathol.* **1996**, *11*, 463–483.
10. Liu, T.; Hu, B.; Choi, Y.Y.; Chung, M.; Ullenbruch, M.; Yu, H.; Lowe, J.B.; Phan, S.H. Notch1 signaling in FIZZ1 induction of myofibroblast differentiation. *Am. J. Pathol.* **2009**, *174*, 1745–1755.
11. Heineke, J.; Auger-Messier, M.; Correll, R.N.; Xu, J.; Benard, M.J.; Yuan, W.; Drexler, H.; Parise, L.V.; Molkentin, J.D. CIB1 is a regulator of pathological cardiac hypertrophy. *Nat. Med.* **2010**, *16*, 872–879.
12. Sisson, T.H.; Ajayi, I.O.; Subbotina, N.; Dodi, A.E.; Rodansky, E.S.; Chibucos, L.N.; Kim, K.K.; Keshamouni, V.G.; White, E.S.; Zhou, Y.; et al. Inhibition of myocardin-related transcription factor/serum response factor signaling decreases lung fibrosis and promotes mesenchymal cell apoptosis. *Am. J. Pathol.* **2015**, *185*, 969–986.
13. Juo, L.-Y.; Liao, W.-C.; Shih, Y.-L.; Yang, B.-Y.; Liu, A.-B.; Yan, Y.-T. HSPB7 interacts with dimerized FLNC and its absence results in progressive myopathy in skeletal muscles. *J. Cell Sci.* **2016**, *129*, 1661–1670.
14. Marguerie, C.; Bunn, C.C.; Beynon, H.L.; Bernstein, R.M.; Hughes, J.M.; So, A.K.; Walport, M.J. Polymyositis, pulmonary fibrosis and autoantibodies to aminoacyl-tRNA synthetase enzymes. *Q. J. Med.* **1990**, *77*, 1019–1038.
15. Rotman, Y.; Koh, C.; Zmuda, J.M.; Kleiner, D.E.; Liang, T.J. NASH CRN The association of genetic variability in patatin-like phospholipase domain-containing protein 3 (PNPLA3) with histological severity of nonalcoholic fatty liver disease. *Hepatology* **2010**, *52*, 894–903.
16. Uchio, K.; Tuchweber, B.; Manabe, N.; Gabbiani, G.; Rosenbaum, J.; Desmoulière, A. Cellular retinol-binding protein-1 expression and modulation during in vivo and in vitro myofibroblastic differentiation of rat hepatic stellate cells and portal fibroblasts. *Lab. Investig.* **2002**, *82*, 619–628.
17. Paun, A.; Haston, C.K. Genomic and genome-wide association of susceptibility to radiation-induced fibrotic lung disease in mice. *Radiother. Oncol. J. Eur. Soc. Ther. Radiol. Oncol.* **2012**, *105*, 350–357.
18. Swiderski, R.E.; Dencoff, J.E.; Floerchinger, C.S.; Shapiro, S.D.; Hunninghake, G.W. Differential expression of extracellular matrix remodeling genes in a murine model of bleomycin-induced pulmonary fibrosis. *Am. J. Pathol.* **1998**, *152*, 821–828.
19. Hashimoto, S.; Amaya, F.; Matsuyama, H.; Ueno, H.; Kikuchi, S.; Tanaka, M.; Watanabe, Y.; Ebina, M.; Ishizaka, A.; Tsukita, S.; et al. Dysregulation of lung injury and repair in moesin-deficient mice treated with intratracheal bleomycin. *Am. J. Physiol. Lung Cell. Mol. Physiol.* **2008**, *295*, L566–L574.
20. Liu, W.; Ding, I.; Chen, K.; Olschowka, J.; Xu, J.; Hu, D.; Morrow, G.R.; Okunieff, P. Interleukin 1β (IL1B) signaling is a critical component of radiation-induced skin fibrosis. *Radiat. Res.* **2006**, *165*, 181–191.
21. Christmann, R.B.; Sampaio-Barros, P.; Stifano, G.; Borges, C.L.; de Carvalho, C.R.; Kairalla, R.; Parra, E.R.; Spira, A.; Simms, R.; Capellozzi, V.L.; et al. Association of Interferon- and transforming growth factor β-regulated genes and macrophage activation with systemic sclerosis-related progressive lung fibrosis. *Arthritis Rheumatol.* **2014**, *66*, 714–725.
22. Park, J.H.; Chung, H.Y.; Kim, M.; Lee, J.H.; Jung, M.; Ha, H. Daumone fed late in life improves survival and reduces hepatic inflammation and fibrosis in mice. *Aging Cell* **2014**, *13*, 709–718.
23. Huang, M.; Sharma, S.; Zhu, L.X.; Keane, M.P.; Luo, J.; Zhang, L.; Burdick, M.D.; Lin, Y.Q.; Dohadwala, M.; Gardner, B.; et al. IL-7 inhibits fibroblast TGF-β production and signaling in pulmonary fibrosis. *J. Clin. Investig.* **2002**, *109*, 931–937.
24. Okada, Y.; Shirai, K.; Reinach, P.S.; Kitano-Izutani, A.; Miyajima, M.; Flanders, K.C.; Jester, J.V.; Tominaga, M.; Saika, S. TRPA1 is required for TGF-β signaling and its loss blocks inflammatory fibrosis in mouse corneal stroma. *Lab. Investig.* **2014**, *94*, 1030–1041.
25. Estep, J.M.; Baranova, A.; Hossain, N.; Elariny, H.; Ankrah, K.; Afendy, A.; Chandhoke, V.; Younossi, Z.M. Expression of cytokine signaling genes in morbidly obese patients with non-alcoholic steatohepatitis and hepatic fibrosis. *Obes. Surg.* **2009**, *19*, 617–624.
26. Shimizu, S.; Gabazza, E.C.; Taguchi, O.; Yasui, H.; Taguchi, Y.; Hayashi, T.; Ido, M.; Shimizu, T.; Nakagaki, T.; Kobayashi, H.; et al. Activated protein C inhibits the expression of platelet-derived growth factor in the lung. *Am. J. Respir. Crit. Care Med.* **2003**, *167*, 1416–1426.

27. Massie, C.; Hudson, M.; Tatibouet, S.; Steele, R.; Huynh, T.; Fritzler, M.J.; Baron, M.; Pineau, C.A.; Canadian Scleroderma Research Group (CSRG). Absence of an association between anti-Ro antibodies and prolonged QTc interval in systemic sclerosis: A multicenter study of 689 patients. *Semin. Arthritis Rheum.* **2014**, *44*, 338–344.
28. Wang, X.; Qian, Y.; Jin, R.; Wo, Y.; Chen, J.; Wang, C.; Wang, D. Effects of TRAP-1-like protein (TLP) gene on collagen synthesis induced by TGF-β/Smad signaling in human dermal fibroblasts. *PLoS ONE* **2013**, *8*, e55899.
29. Komada, T.; Usui, F.; Shirasuna, K.; Kawashima, A.; Kimura, H.; Karasawa, T.; Nishimura, S.; Sagara, J.; Noda, T.; Taniguchi, S.I.; et al. ASC in renal collecting duct epithelial cells contributes to inflammation and injury after unilateral ureteral obstruction. *Am. J. Pathol.* **2014**, *184*, 1287–1298.
30. Younossi, Z.M.; Afendy, A.; Stepanova, M.; Hossain, N.; Younossi, I.; Ankrah, K.; Gramlich, T.; Baranova, A. Gene expression profile associated with superimposed non-alcoholic fatty liver disease and hepatic fibrosis in patients with chronic hepatitis C. *Liver Int.* **2009**, *29*, 1403–1412.
31. White, D.L.; Liu, Y.; Garcia, J.; El-Serag, H.B.; Jiao, L.; Tsavachidis, S.; Franco, L.M.; Lee, J.-S.; Tavakoli-Tabasi, S.; Moore, D.; et al. Sex hormone pathway gene polymorphisms are associated with risk of advanced hepatitis C-related liver disease in males. *Int. J. Mol. Epidemiol. Genet.* **2014**, *5*, 164–176.
32. Wang, W.; Liu, H.; Dai, X.; Fang, S.; Wang, X.; Zhang, Y.; Yao, H.; Zhang, X.; Chao, J. p53/PUMA expression in human pulmonary fibroblasts mediates cell activation and migration in silicosis. *Sci. Rep.* **2015**, *5*, 16900.
33. Moschen, A.R.; Fritz, T.; Clouston, A.D.; Rebhan, I.; Bauhofer, O.; Barrie, H.D.; Powell, E.E.; Kim, S.-H.; Dinarello, C.A.; Bartenschlager, R.; et al. Interleukin-32: A new proinflammatory cytokine involved in hepatitis C virus-related liver inflammation and fibrosis. *Hepatology* **2011**, *53*, 1819–1829.
34. Yee, L.J.; Tang, Y.-M.; Kleiner, D.E.; Wang, D.; Im, K.; Wahed, A.; Tong, X.; Rhodes, S.; Su, X.; Whelan, R.M.; et al. Myxovirus-1 and protein kinase haplotypes and fibrosis in chronic hepatitis C virus. *Hepatology* **2007**, *46*, 74–83.
35. Sohn, S.-H.; Lee, J.M.; Park, S.; Yoo, H.; Kang, J.W.; Shin, D.; Jung, K.-H.; Lee, Y.-S.; Cho, J.; Bae, H. The inflammasome accelerates radiation-induced lung inflammation and fibrosis in mice. *Environ. Toxicol. Pharmacol.* **2015**, *39*, 917–926.
36. Martin, T.R.; Hagimoto, N.; Nakamura, M.; Matute-Bello, G. Apoptosis and epithelial injury in the lungs. *Proc. Am. Thorac. Soc.* **2005**, *2*, 214–220.
37. Antoniou, K.M.; Tzouvelekis, A.; Alexandrakis, M.G.; Sfiridaki, K.; Tsiligianni, I.; Rachiotis, G.; Tzanakis, N.; Bouros, D.; Milic-Emili, J.; Siafakas, N.M. Different angiogenic activity in pulmonary sarcoidosis and idiopathic pulmonary fibrosis. *Chest* **2006**, *130*, 982–988.
38. Sigdel, T.K.; Bestard, O.; Tran, T.Q.; Hsieh, S.-C.; Roedder, S.; Damm, I.; Vincenti, F.; Sarwal, M.M. A Computational Gene Expression Score for Predicting Immune Injury in Renal Allografts. *PLoS ONE* **2015**, *10*, e0138133.
39. Shi, J.; Wang, H.; Guan, H.; Shi, S.; Li, Y.; Wu, X.; Li, N.; Yang, C.; Bai, X.; Cai, W.; et al. IL10 inhibits starvation-induced autophagy in hypertrophic scar fibroblasts via cross talk between the IL10-IL10R-STAT3 and IL10-AKT-mTOR pathways. *Cell Death Dis.* **2016**, *7*, e2133.
40. Bièche, I.; Asselah, T.; Laurendeau, I.; Vidaud, D.; Degot, C.; Paradis, V.; Bedossa, P.; Valla, D.-C.; Marcellin, P.; Vidaud, M. Molecular profiling of early stage liver fibrosis in patients with chronic hepatitis C virus infection. *Virology* **2005**, *332*, 130–144.
41. Keane, M.P.; Belperio, J.A.; Moore, T.A.; Moore, B.B.; Arenberg, D.A.; Smith, R.E.; Burdick, M.D.; Kunkel, S.L.; Strieter, R.M. Neutralization of the CXC chemokine, macrophage inflammatory protein-2, attenuates bleomycin-induced pulmonary fibrosis. *J. Immunol.* **1999**, *162*, 5511–5518.
42. Maluf, D.G.; Mas, V.R.; Archer, K.J.; Yanek, K.; Gibney, E.M.; King, A.L.; Cotterell, A.; Fisher, R.A.; Posner, M.P. Molecular pathways involved in loss of kidney graft function with tubular atrophy and interstitial fibrosis. *Mol. Med. Camb. Mass* **2008**, *14*, 276–285.
43. Bozyk, P.D.; Moore, B.B. Prostaglandin E2 and the pathogenesis of pulmonary fibrosis. *Am. J. Respir. Cell Mol. Biol.* **2011**, *45*, 445–452.
44. Herro, R.; Croft, M. The control of tissue fibrosis by the inflammatory molecule LIGHT (TNF Superfamily member 14). *Pharmacol. Res.* **2016**, *104*, 151–155.

45. Domagalski, K.; Pawłowska, M.; Kozielewicz, D.; Dybowska, D.; Tretyn, A.; Halota, W. The Impact of IL28B Genotype and Liver Fibrosis on the Hepatic Expression of *IP10*, *IFI27*, *ISG15*, and *MX1* and Their Association with Treatment Outcomes in Patients with Chronic Hepatitis C. *PLoS ONE* **2015**, *10*, e0130899.
46. Liu, X.; Gao, J.; Xia, Q.; Lu, T.; Wang, F. Increased mortality and aggravation of heart failure in liver X receptor-α knockout mice after myocardial infarction. *Heart Vessels* **2016**, *31*, 1370–1379.
